# Selective cTACE to a segment target hepatocellular carcinoma lesion during atezolizumab plus bevacizumab therapy achieving delayed hypovascularization and shrinkage of non-target intrahepatic lesions: a case report

**DOI:** 10.1093/omcr/omag171

**Published:** 2026-09-08

**Authors:** Hidenobu Hara, Hiroaki Matsumoto, Ami Yoshinaga, Hikari Ishii, Kei Onodera, Yoko Henta, Risa Katsumata, Ryosuke Fujii, Harujiro Yamamoto, Tomohisa Ashikawa, Kazuomi Sakaki, Shiori Ito, Takehito Asakawa, Kohei Yoshino, Shinya Sakita

**Affiliations:** Department of Gastroenterology, Yokohama City Minato Red Cross Hospital, 3-12-1 Shin-yamashita, Naka-ku, Yokohama, Kanagawa 231-8682, Japan; Department of Gastroenterology, Yokohama City Minato Red Cross Hospital, 3-12-1 Shin-yamashita, Naka-ku, Yokohama, Kanagawa 231-8682, Japan; Department of Gastroenterology, Yokohama City Minato Red Cross Hospital, 3-12-1 Shin-yamashita, Naka-ku, Yokohama, Kanagawa 231-8682, Japan; Department of Gastroenterology, Yokohama City Minato Red Cross Hospital, 3-12-1 Shin-yamashita, Naka-ku, Yokohama, Kanagawa 231-8682, Japan; Department of Gastroenterology, Yokohama City Minato Red Cross Hospital, 3-12-1 Shin-yamashita, Naka-ku, Yokohama, Kanagawa 231-8682, Japan; Department of Gastroenterology, Yokohama City Minato Red Cross Hospital, 3-12-1 Shin-yamashita, Naka-ku, Yokohama, Kanagawa 231-8682, Japan; Department of Gastroenterology, Yokohama City Minato Red Cross Hospital, 3-12-1 Shin-yamashita, Naka-ku, Yokohama, Kanagawa 231-8682, Japan; Department of Gastroenterology, Yokohama City Minato Red Cross Hospital, 3-12-1 Shin-yamashita, Naka-ku, Yokohama, Kanagawa 231-8682, Japan; Department of Gastroenterology, Yokohama City Minato Red Cross Hospital, 3-12-1 Shin-yamashita, Naka-ku, Yokohama, Kanagawa 231-8682, Japan; Department of Gastroenterology, Yokohama City Minato Red Cross Hospital, 3-12-1 Shin-yamashita, Naka-ku, Yokohama, Kanagawa 231-8682, Japan; Department of Gastroenterology, Yokohama City Minato Red Cross Hospital, 3-12-1 Shin-yamashita, Naka-ku, Yokohama, Kanagawa 231-8682, Japan; Department of Gastroenterology, Yokohama City Minato Red Cross Hospital, 3-12-1 Shin-yamashita, Naka-ku, Yokohama, Kanagawa 231-8682, Japan; Department of Gastroenterology, Yokohama City Minato Red Cross Hospital, 3-12-1 Shin-yamashita, Naka-ku, Yokohama, Kanagawa 231-8682, Japan; Department of Gastroenterology, Yokohama City Minato Red Cross Hospital, 3-12-1 Shin-yamashita, Naka-ku, Yokohama, Kanagawa 231-8682, Japan; Department of Gastroenterology, Yokohama City Minato Red Cross Hospital, 3-12-1 Shin-yamashita, Naka-ku, Yokohama, Kanagawa 231-8682, Japan

**Keywords:** hepatocellular carcinoma, atezolizumab, bevacizumab, cTACE, mRECIST, intrahepatic progression

## Abstract

Atezolizumab plus bevacizumab (Atez/Bev) is the standard first-line systemic therapy for unresectable hepatocellular carcinoma (HCC). We report HCC with intrahepatic progression during Atez/Bev in which selective conventional transarterial chemoembolization (cTACE) to a single lesion was followed by non-target lesion shrinkage. An 82-year-old woman with metabolic dysfunction-associated steatotic liver disease was diagnosed with BCLC stage B hepatocellular carcinoma in segments 5/7. After TACE, residual viable tumor persisted in segment 5, and Atez/Bev was initiated. After 4 cycles, a new intrahepatic lesion appeared in segment 8, and after 7 cycles, lesions in segments 5/7/8 enlarged, meeting the mRECIST progressive disease criteria. Systemic therapy continued, and selective cTACE for the segment 5 lesion was added. At 3 months post-cTACE, segment 5 viability decreased, but overall mRECIST remained progressive disease. At 6 months (November 20XX + 1), arterial enhancement in segment 5 resolved and non-target segment 7/8 lesions became hypovascular and shrank, achieving partial response.

## Introduction

Atezolizumab plus bevacizumab (Atez/Bev) is the standard first-line systemic therapy for cases of unresectable hepatocellular carcinoma (HCC) [1, 2]. In practice, intrahepatic lesions can still progress during systemic therapy, while the role of additional locoregional therapy during continuing systemic treatment is poorly established. Transarterial chemoembolization (TACE) can ensure intrahepatic tumor control, but repeated or extensive embolization can compromise liver function; therefore, the careful selection of candidates and treatment extent is important [3].

Herein, we report the case of a patient with HCC who showed intrahepatic progression during Atez/Bev treatment, in whom selective conventional TACE (cTACE) to a single target lesion was followed by delayed improvement of the non-target intrahepatic lesions.

## Case report

**Figure 1:**
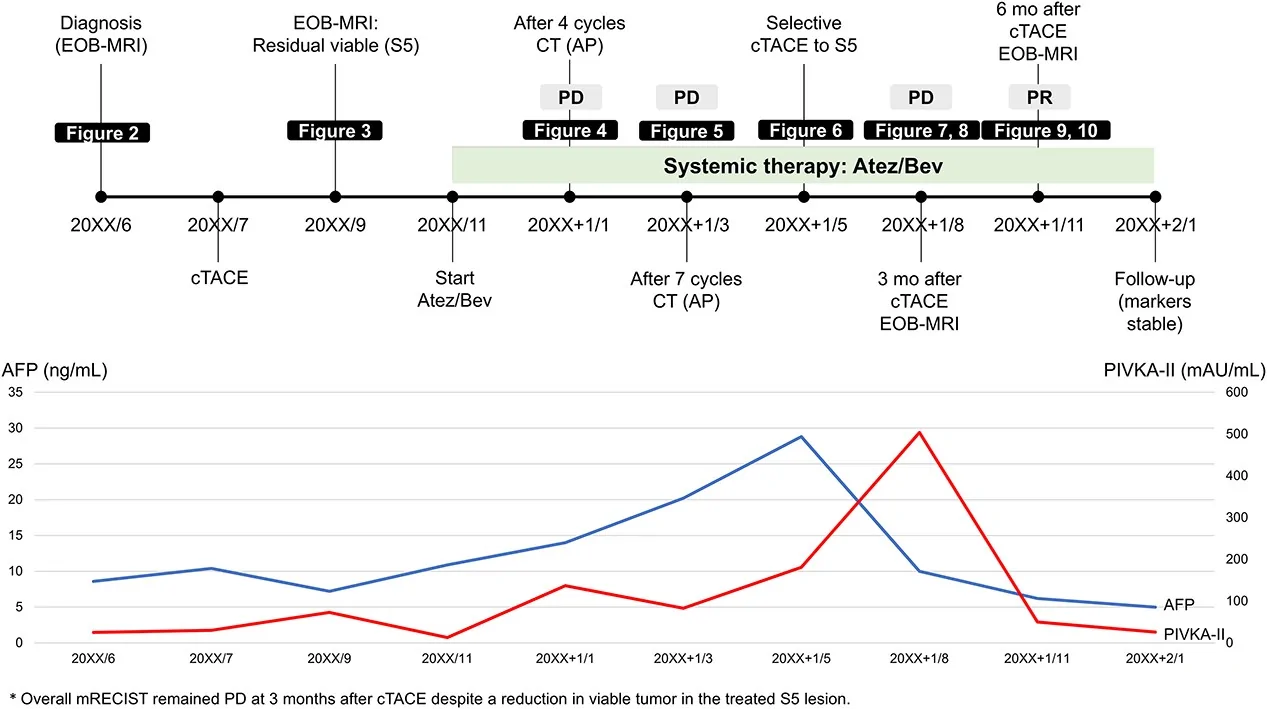
Overview of the patient’s clinical course, imaging assessments, and tumor marker trends. The timeline shows key imaging time points and treatments during atezolizumab plus bevacizumab therapy, including the performance of selective cTACE to the segment 5 lesion. AFP and PIVKA-II are plotted over time. ^*^Overall mRECIST remained ‘progressive disease’ at 3 months after cTACE despite a reduction in viable tumor in the treated S5 lesion.

**Figure 2:**
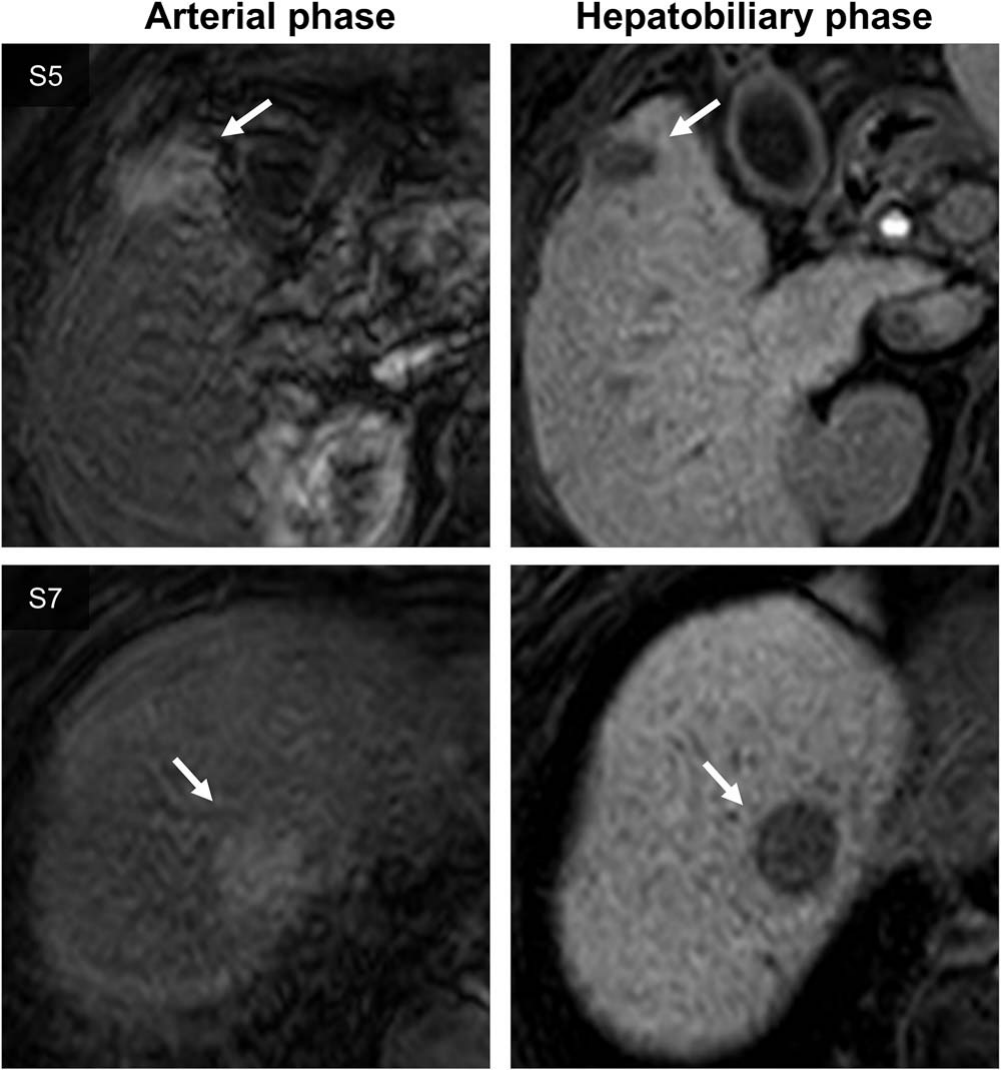
Baseline EOB-MRI findings of hepatocellular carcinoma tumors in segments 5 and 7. Arterial-phase and hepatobiliary-phase images are shown. The arrows indicate the lesions.

**Figure 3:**
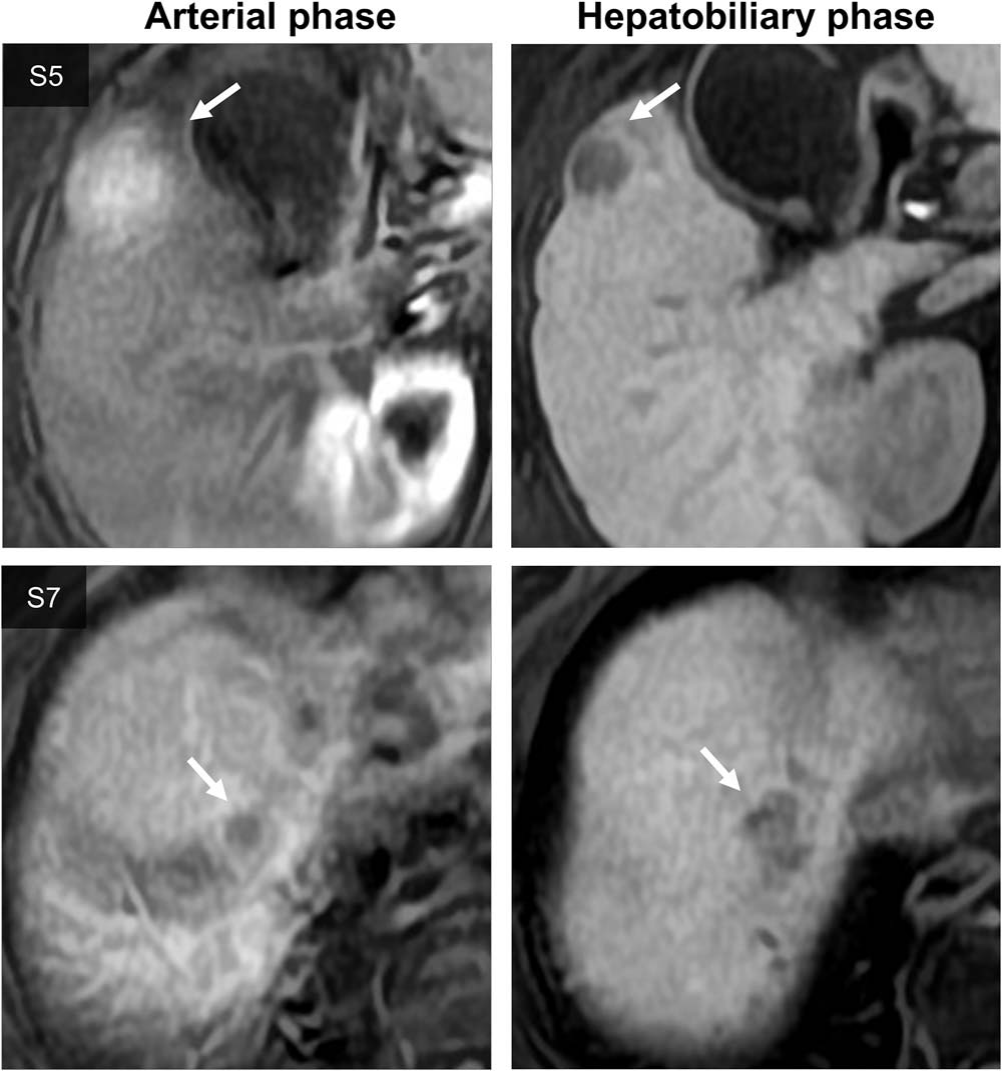
Arterial- and hepatobiliary-phase images obtained after transarterial chemoembolization. Residual arterial enhancement persisted in the segment 5 lesion, indicating residual viable tumor; the segment 7 lesion is shown for comparison.

The patient, an 82-year-old woman with metabolic dysfunction-associated steatotic liver disease, presented to undergo evaluation of liver tumors. Liver function was preserved (Child-Pugh A [5 points], modified albumin-bilirubin grade 1). Her clinical course and treatment timeline are summarized in Fig. 1. In June 20XX, EOB-MRI revealed HCC in segments 5 (16 mm) and 7 (23 mm) (Fig. 2), consistent with BCLC stage B disease. cTACE was performed in July 20XX; however, follow-up EOB-MRI in September 20XX demonstrated residual viable tumor in the segment 5 lesion, and the treatment response was considered insufficient (Fig. 3). Atez/Bev was therefore started in November 20XX.

**Figure 4:**
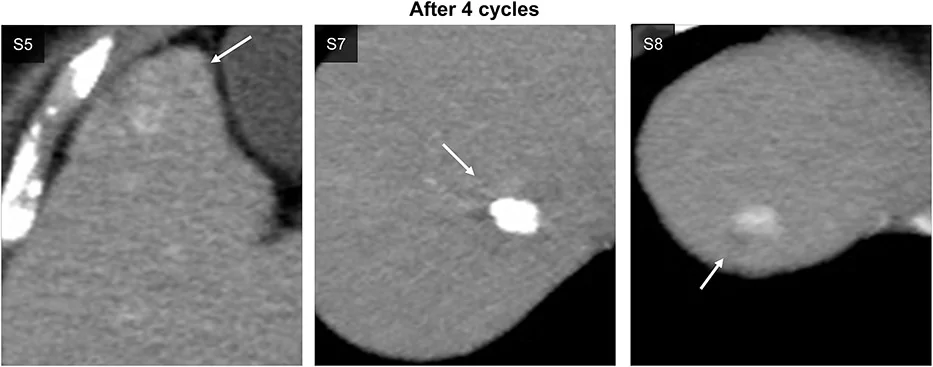
Arterial-phase CT conducted after 4 cycles of atezolizumab plus bevacizumab. The pre-existing lesions (segments 5 and 7) show no marked change, while a new intrahepatic lesion can be observed in segment 8 (arrows).

**Figure 5:**
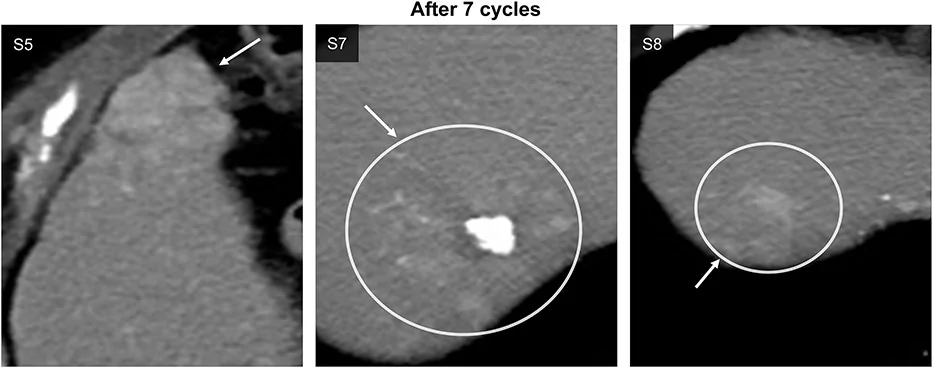
Arterial-phase CT conducted after 7 cycles of atezolizumab plus bevacizumab. Lesions in segments 5, 7, and 8 show progression with enlargement and/or expansion of enhancing areas, consistent with progressive disease by mRECIST.

At the first radiologic assessment conducted after 4 cycles (January 20XX + 1), arterial-phase computed tomography (CT) showed no marked change in the pre-existing lesions, but a new intrahepatic lesion had developed in segment 8 (Fig. 4). Although this met progressive disease criteria according to the modified Response Evaluation Criteria in Solid Tumors (mRECIST) guideline [4], treatment was continued because this was the first assessment and the pre-existing lesions showed minimal change. After 7 cycles (March 20XX + 1), arterial-phase CT showed enlargement of the lesions in segments 5/7/8, with expansion of enhancing areas; the overall response was classified as progressive disease by mRECIST (Fig. 5). During systemic therapy, bevacizumab was withheld during cycles 5, 6, and 7 (a total of 63 days) and again during cycle 9 (21 days) because of grade 2 proteinuria, while atezolizumab was continued. In addition, bevacizumab was withheld in cycle 9 immediately before locoregional therapy. The cumulative duration of bevacizumab interruption was 84 days (12 weeks).

**Figure 6:**
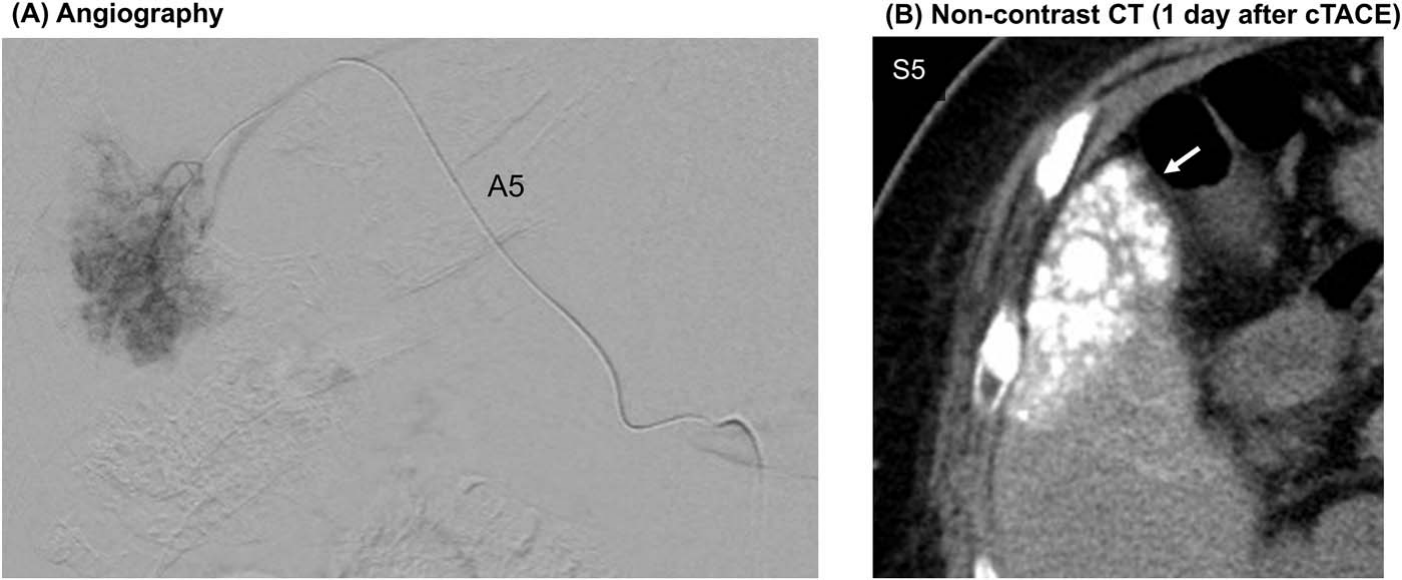
Selective conventional transarterial chemoembolization for the segment 5 lesion. (A) Angiography showing tumor staining supplied by A5. (B) Non-contrast CT (1 day after cTACE), confirming Lipiodol deposition in the treated lesion.

**Figure 7:**
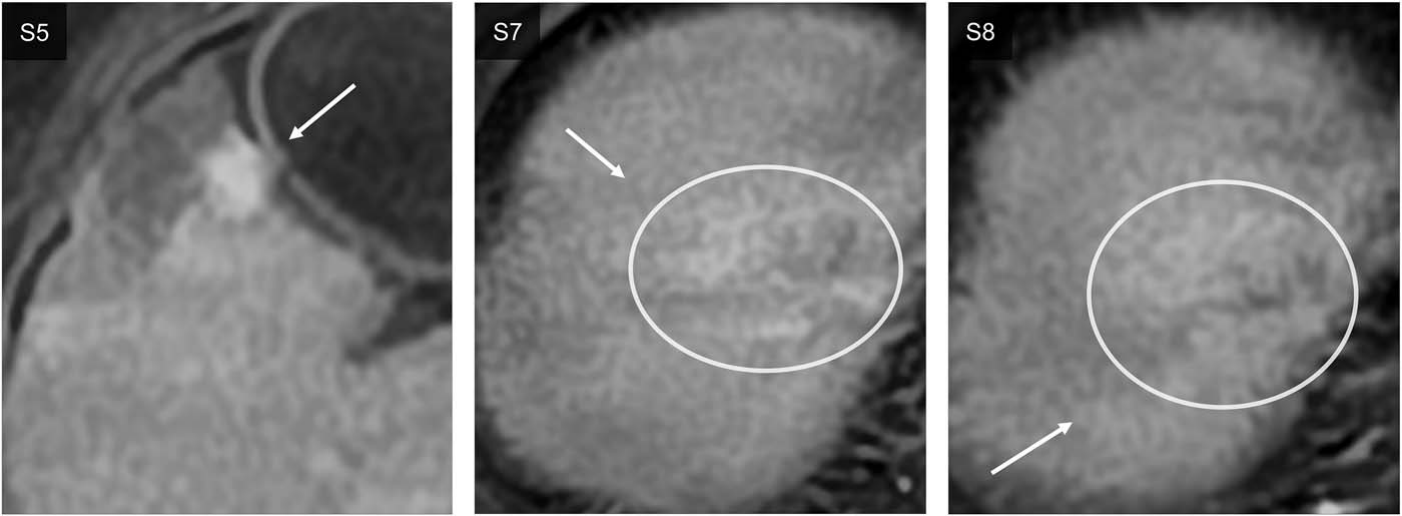
EOB-MRI at 3 months following conventional transarterial chemoembolization, in the arterial phase. Residual arterial enhancement persists in the treated segment 5 lesion, while non-target lesions in segments 7 and 8 show no clear improvement.

**Figure 8:**
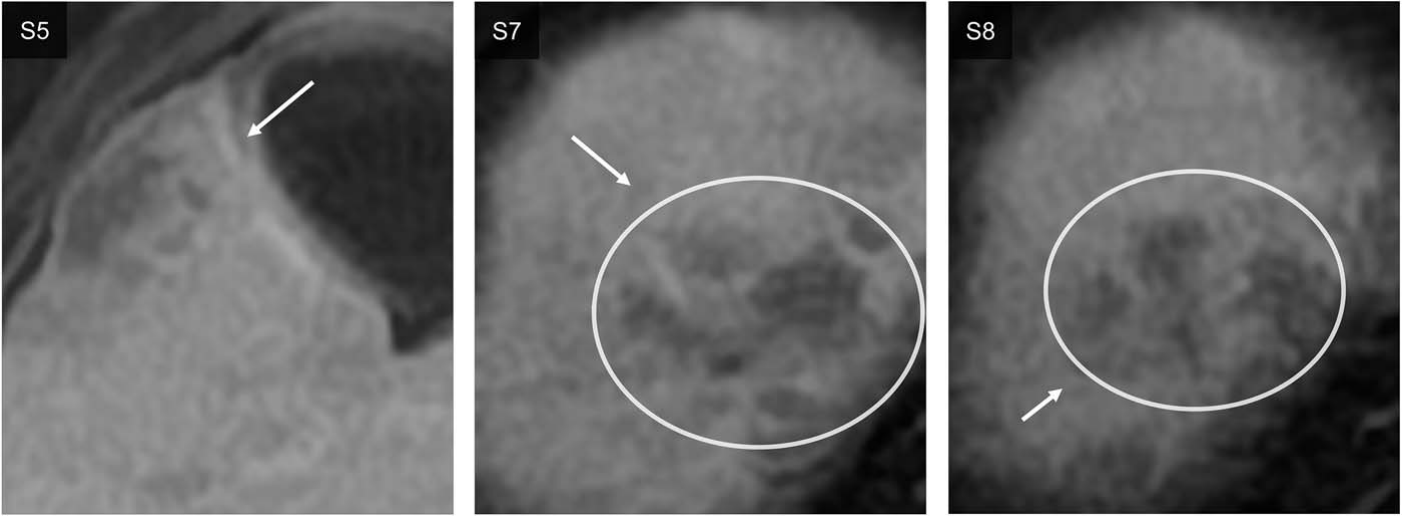
EOB-MRI at 3 months after conventional transarterial chemoembolization, in the hepatobiliary phase. The treated segment 5 lesion and non-target lesions in segments 7 and 8 remain visible.

**Figure 9:**
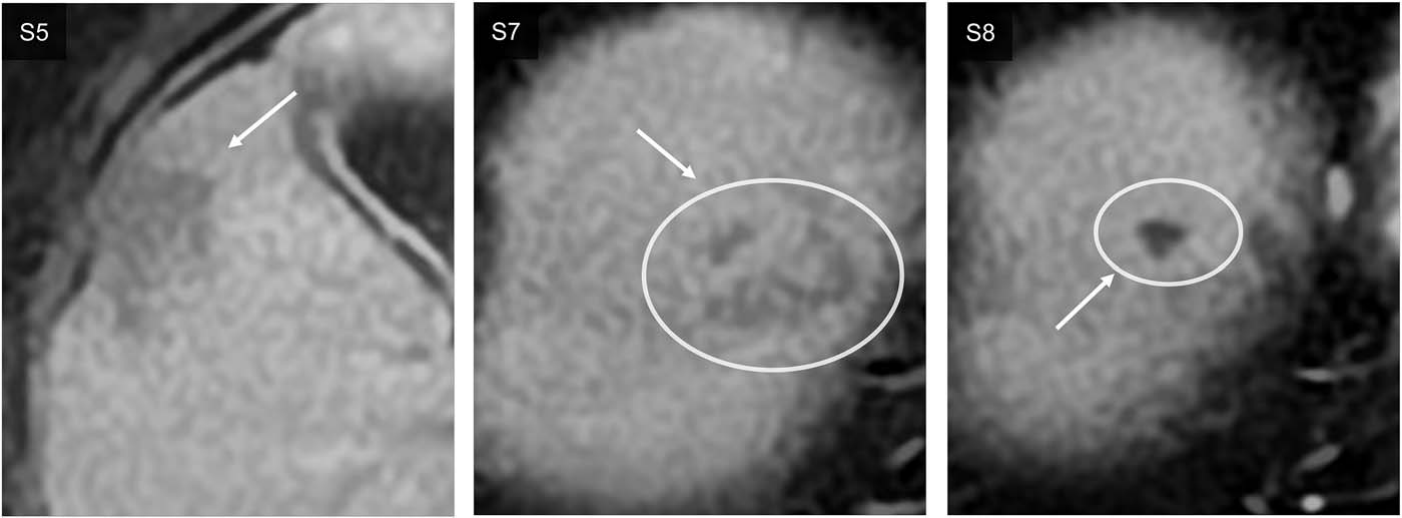
EOB-MRI at 6 months after conventional transarterial chemoembolization, in the arterial phase. Arterial enhancement in the treated segment 5 lesion had resolved, and non-target lesions in segments 7 and 8 became hypovascular.

**Figure 10:**
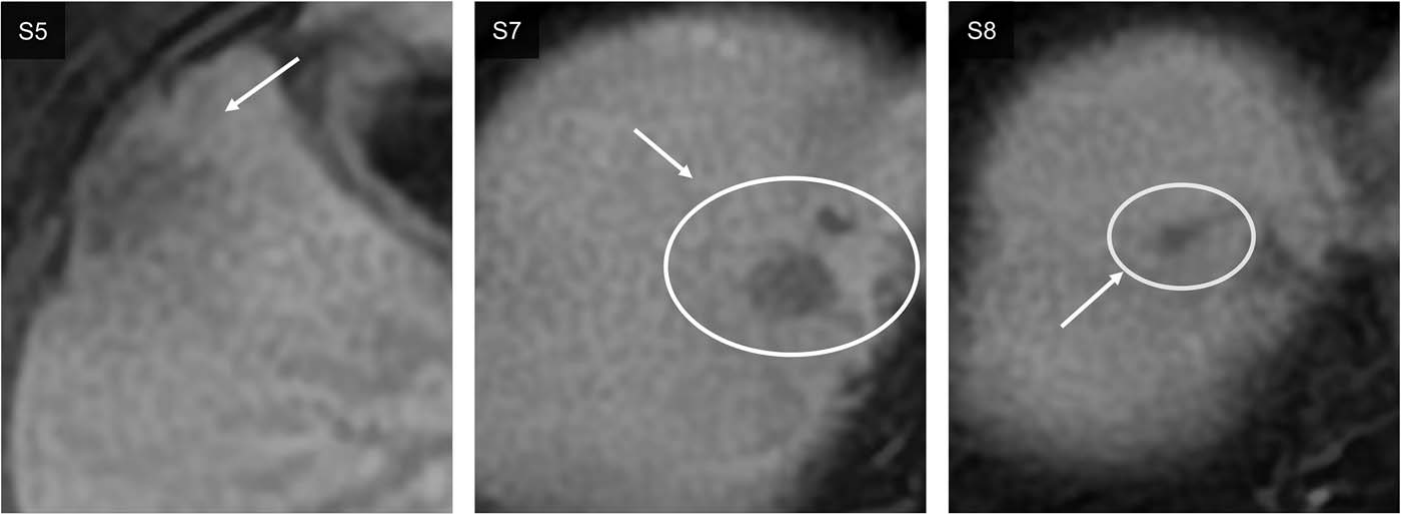
EOB-MRI at 6 months after conventional transarterial chemoembolization: Hepatobiliary phase. The non-target lesions in segments 7 and 8 decreased in size compared with the 3-month assessment, consistent with partial response by mRECIST.

To preserve liver function and enable continued systemic therapy, selective cTACE was added only to the segment 5 lesion in May 20XX + 1, with the following parameters: epirubicin 20 mg and Lipiodol 2 mL, followed by gelatin sponge embolization, with cone-beam CT confirming deposition in the target lesion (Fig. 6). Atez/Bev was resumed from cycle 10 in July 20XX + 1 and continued thereafter. At 3 months post-cTACE (August 20XX + 1), gadolinium-ethoxybenzyl-diethylenetriamine pentaacetic acid magnetic resonance imaging (EOB-MRI) showed that arterial enhancement in segment 5 was limited to a small peripheral nodular focus, suggesting reduced viable tumor, whereas the non-target lesions in segments 7 and 8 showed no clear improvement. Accordingly, the overall mRECIST response remained progressive disease (Figs 7 and 8). At 6 months (November 20XX + 1), arterial enhancement in segment 5 had disappeared, and non-target lesions in segments 7 and 8 became hypovascular and shrank, meeting the partial response criteria by mRECIST (Figs 9 and 10). Adverse events included proteinuria (grade 2) and mild pruritus (grade 1).

## Discussion

This case illustrates a pragmatic strategy for intrahepatic progression during Atez/Bev therapy: adding selective cTACE to a single target lesion while continuing systemic therapy. Although the overall mRECIST response remained progressive disease at 3 months, imaging at 6 months demonstrated disappearance of arterial enhancement in the treated lesion and delayed hypovascularization and shrinkage of non-target lesions, meeting partial response criteria.

A notable feature of this case was the delayed hypovascularization and shrinkage of non-target lesions in segments 7 and 8, despite cTACE being performed only on segment 5. Several alternative explanations should be considered. First, delayed responses to Atez/Bev have been reported, and the response observed at 6 months in the present case could reflect a delayed effect of systemic therapy rather than a consequence of selective cTACE [5]. In the GO30140 study, 19% of confirmed responders (7/37) demonstrated delayed responses, with a median time to response of 8.3 months (range 5.4–16.4 months). In our patient, 13 cycles were administered over approximately 9.75 months from initiation to the 6-month post-cTACE assessment, which falls within this reported window. Second, an immune-mediated systemic effect triggered by locoregional therapy (an ‘abscopal-like’ phenomenon) is biologically plausible; however, evidence in HCC remains limited and at times contradictory. Therefore, causality cannot be inferred from a single case [6]. Third, bevacizumab interruption and subsequent resumption may have influenced tumor vascularity and radiologic appearance and should be considered an important confounder when interpreting the temporal association between cTACE and later imaging changes. Bevacizumab interruption and resumption may also have influenced arterial enhancement patterns, representing an important confounder in an mRECIST-based interpretation. As bevacizumab inhibits VEGF-mediated angiogenesis, its withdrawal may permit vascular rebound, whereas resumption can re-establish anti-angiogenic effects—potentially modifying tumor vascularity and arterial enhancement independent of true changes in viable tumor burden. Prior studies have reported modulation of tumor vascularity and imaging findings associated with bevacizumab interruption/resumption; therefore, given the temporal overlap between bevacizumab withholding (cycles 5–7 and 9; cumulative 84 days) and subsequent imaging changes, causal attribution of the non-target lesion response solely to selective cTACE should be avoided [7, 8]. While mechanistic interpretation is limited in a single case, this course is clinically informative in that selective cTACE to a single target lesion can be performed to preserve liver function while continuing systemic therapy, and delayed improvement of non-target intrahepatic lesions may be observed.

Combination and sequencing strategies of locoregional therapy and systemic therapy are increasingly discussed for intermediate-stage HCC. In addition to early-phase data, phase III evidence has recently shown improved progression-free survival when immunotherapy-based regimens are combined with TACE in patients with unresectable HCC amenable to embolization. Real-world emulation studies have also supported the feasibility of TACE combined with immunotherapy and anti-VEGF–based therapy [9, 10].
